# 416. Poor clinical outcomes of COVID-19 patients receiving rituximab during the Omicron era

**DOI:** 10.1093/ofid/ofad500.486

**Published:** 2023-11-27

**Authors:** Chan Mi Lee, Chang Kyung Kang, Hyeon Jae Jo, Pyoeng Gyun Choe, Nam Joong Kim, Wan Beom Park, Myoung-don Oh

**Affiliations:** Seoul National University College of Medicine, Seoul, Seoul-t'ukpyolsi, Republic of Korea; Seoul National University College of Medicine, Seoul, Seoul-t'ukpyolsi, Republic of Korea; Seoul National University College of Medicine, Seoul, Seoul-t'ukpyolsi, Republic of Korea; Seoul National University College of Medicine, Seoul, Seoul-t'ukpyolsi, Republic of Korea; Seoul National University College of Medicine, Seoul, Seoul-t'ukpyolsi, Republic of Korea; Seoul National University College of Medicine, Seoul, Seoul-t'ukpyolsi, Republic of Korea; Department of Internal Medicine, Seoul National University College of Medicine, Seoul, Korea, Seoul, Seoul-t'ukpyolsi, Republic of Korea

## Abstract

**Background:**

Rituximab (RTX), a widely used anti-CD20 monoclonal antibody, has been suggested as a risk factor for poor outcomes of coronavirus disease 2019 (COVID-19). However, it remains largely unknown whether COVID-19 outcomes remain worse in patients receiving RTX compared to those not being treated with RTX during the Omicron-dominant period, when patient fatality has significantly decreased compared to the Delta-dominant period. Therefore, we aimed to investigate the outcomes of COVID-19 patients receiving RTX during the Omicron-dominant period.

**Methods:**

Among all laboratory-confirmed COVID-19 patients who were admitted to Seoul National University Hospital from February 2022 to January 2023, those who had received RTX before diagnosis of COVID-19 were included in the RTX group, while those who had the same underlying diseases but had not received RTX were included in the non-RTX group. The COVID-19-related outcomes were compared between the RTX and non-RTX groups. Multivariate logistic regression analyses were used to identify factors associated with severe to critical COVID-19 and COVID-19-related mortality.

**Results:**

The proportion of severe to critical COVID-19 and COVID-19-related mortality were significantly higher in the RTX group than in the non-RTX group (41.9% *vs.* 28.3%, *P* = 0.030; 11.8% *vs.* 2.8%, *P* = 0.005). RTX therapy (adjusted odds ratio [aOR] 2.21, 95% confidence interval [CI] 1.21–4.04, *P* = 0.010) and chronic kidney disease (aOR 3.28, 95% CI 1.56–6.91, *P* = 0.002) were independent risk factors for severe to critical COVID-19, whereas being effectively vaccinated (aOR 0.47, 95% CI 0.25–0.85, *P* = 0.013) was a protective factor. In addition, only RTX therapy was independently associated with COVID-19-related mortality (aOR 4.03, 95% CI 1.17–13.86, *P* = 0.027).

**Figure:**
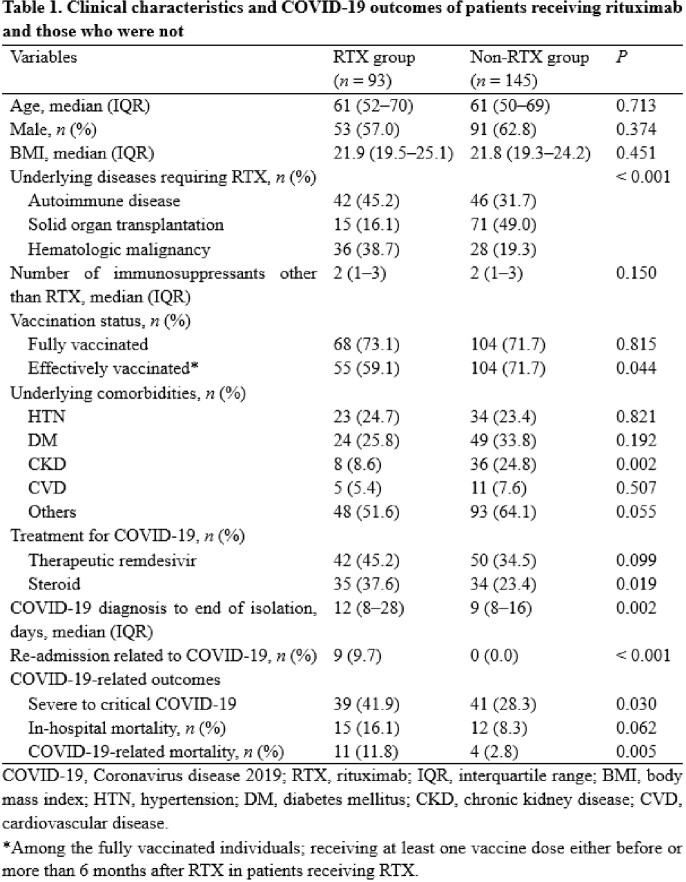

**Figure:**
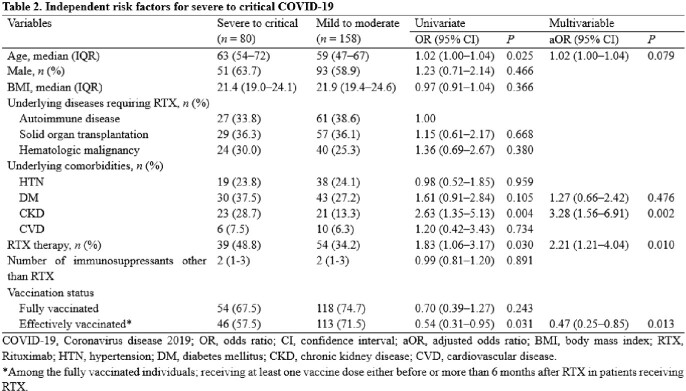

**Conclusion:**

RTX therapy was associated with poor clinical outcomes of COVID-19, even in the Omicron-dominant period. This suggests that RTX should be used with caution even during the Omicron-dominant period.

**Disclosures:**

**All Authors**: No reported disclosures

